# Cardiac Energetics in Patients With Aortic Stenosis and Preserved Versus Reduced Ejection Fraction

**DOI:** 10.1161/CIRCULATIONAHA.119.043450

**Published:** 2020-05-22

**Authors:** Mark A. Peterzan, William T. Clarke, Craig A. Lygate, Hannah A. Lake, Justin Y.C. Lau, Jack J. Miller, Errin Johnson, Jennifer J. Rayner, Moritz J. Hundertmark, Rana Sayeed, Mario Petrou, George Krasopoulos, Vivek Srivastava, Stefan Neubauer, Christopher T. Rodgers, Oliver J. Rider

**Affiliations:** 1 Oxford Centre for Clinical Magnetic Resonance Research, Division of Cardiovascular Medicine (M.A.P., J.Y.C.L., J.J.M., J.J.R., M.J.H., S.N., O.J.R.), University of Oxford, United Kingdom.; 2 Wellcome Centre for Integrative Neuroimaging, FMRIB, Nuffield Department of Clinical Neurosciences (W.T.C.), University of Oxford, United Kingdom.; 3 Division of Cardiovascular Medicine, Radcliffe Department of Medicine (H.A.L.), University of Oxford, United Kingdom.; 4 Dunn School of Pathology (E.J.), University of Oxford, United Kingdom.; 5 Department of Cardiothoracic Surgery, Oxford Heart Centre, John Radcliffe Hospital, United Kingdom (R.S., G.K., V.S.).; 6 Department of Cardiothoracic Surgery, Royal Brompton and Harefield National Health Service Foundation Trust, London, United Kingdom (M.P.).; 7 Wolfson Brain Imaging Centre, University of Cambridge, United Kingdom (C.T.R.).

**Keywords:** aortic valve stenosis, creatine kinase, heart failure, magnetic resonance spectroscopy, metabolism

## Abstract

Why some but not all patients with severe aortic stenosis (SevAS) develop otherwise unexplained reduced systolic function is unclear. We investigate the hypothesis that reduced creatine kinase (CK) capacity and flux is associated with this transition.

**Methods:**

We recruited 102 participants to 5 groups: moderate aortic stenosis (ModAS) (n=13), SevAS, left ventricular (LV) ejection fraction ≥55% (SevAS-preserved ejection fraction, n=37), SevAS, LV ejection fraction <55% (SevAS-reduced ejection fraction, n=15), healthy volunteers with nonhypertrophied hearts with normal systolic function (normal healthy volunteer, n=30), and patients with nonhypertrophied, non–pressure-loaded hearts with normal systolic function undergoing cardiac surgery and donating LV biopsy (non–pressure-loaded heart biopsy, n=7). All underwent cardiac magnetic resonance imaging and ^31^P magnetic resonance spectroscopy for myocardial energetics. LV biopsies (AS and non–pressure-loaded heart biopsy) were analyzed for CK total activity, CK isoforms, citrate synthase activity, and total creatine. Mitochondria-sarcomere diffusion distances were calculated by using serial block-face scanning electron microscopy.

**Results:**

In the absence of failure, CK flux was lower in the presence of AS (by 32%, *P*=0.04), driven primarily by reduction in phosphocreatine/ATP (by 17%, *P*<0.001), with CK *k*_*f*_ unchanged (*P*=0.46). Although lowest in the SevAS-reduced ejection fraction group, CK flux was not different from the SevAS-preserved ejection fraction group (*P*>0.99). Accompanying the fall in CK flux, total CK and citrate synthase activities and the absolute activities of mitochondrial-type CK and CK-MM isoforms were also lower (*P*<0.02, all analyses). Median mitochondria-sarcomere diffusion distances correlated well with CK total activity (*r*=0.86, *P*=0.003).

**Conclusions:**

Total CK capacity is reduced in SevAS, with median values lowest in those with systolic failure, consistent with reduced energy supply reserve. Despite this, in vivo magnetic resonance spectroscopy measures of resting CK flux suggest that ATP delivery is reduced earlier, at the moderate AS stage, where LV function remains preserved. These findings show that significant energetic impairment is already established in moderate AS and suggest that a fall in CK flux is not by itself a necessary cause of transition to systolic failure. However, because ATP demands increase with AS severity, this could increase susceptibility to systolic failure. As such, targeting CK capacity and flux may be a therapeutic strategy to prevent and treat systolic failure in AS.

## Abstract 2

Supplemental Digital Content is available in the text.

**Clinical Perspective**

**What Is New?**

- Total creatine kinase (CK) activity is reduced in severe aortic stenosis (SevAS) with preserved systolic function and is lowest in AS with systolic failure.
- At rest, CK flux is not different between AS with preserved systolic function and AS with systolic failure, and is reduced in moderate AS.
- Reduced CK activity is associated with CK isoform redistribution and cytoarchitectural reorganization with a reduction in mitochondrial-sarcomere (ATP) diffusion distance.

**What Are the Clinical Implications?**

- In the presence of SevAS, reduced CK flux is unlikely to be sufficient alone to cause systolic failure, but may predispose to the transition to systolic failure in some patients.
- Targeting CK capacity and flux may be a therapeutic strategy to prevent/treat systolic failure in AS.

In patients with severe aortic stenosis (SevAS), reductions in left ventricular ejection fraction (LVEF) increase mortality irrespective of symptoms. Although most patients present with preserved systolic function, ~30% of asymptomatic patients have borderline LVEF (50%–59%),^1–3^ and 15% of those undergoing surgical aortic valve replacement have reduced LVEF (LVEF <50%).^2^ At present, identifying all individuals who develop an otherwise unexplained decline in LVEF in the face of pressure overload is not possible,^4^ and why some but not all patients with SevAS develop otherwise unexplained reduced LVEF is unclear.

Given the direct coupling of ATP metabolism and contractile function, metabolic reserve could represent an early and specific marker of vulnerability to failure in the face of pressure overload. In the heart, ATP delivery can occur through the creatine kinase (CK) system, which catalyzes the following reversible reaction: Phosphocreatine + ADP + H^+^ ↔ Creatine + ATP. However, in addition to this shuttle function, the CK system can also act as a buffer to dampen changes in ATP and ADP levels. Although the relative importance of these 2 functions is unclear, the CK system is important to preserve the free energy of ATP hydrolysis in the cytosol (Figure 1), a key determinant of contractile function, and it is established that CK capacity (estimated by CK Total Activity×[Total Creatine])^5^ represents an important metabolic reserve, in particular, at high workloads, and correlates closely with contractile reserve.^5–7^

**Figure 1.**
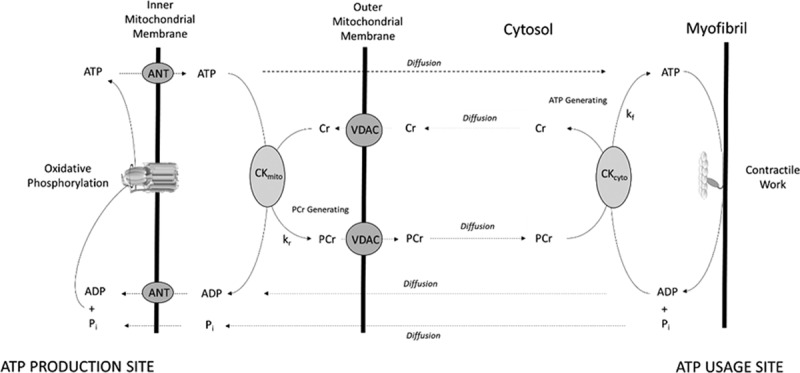
**A schematic of adenine nucleotide transfer pathways in the heart**. This figure shows simple diffusion of ATP, ADP, and inorganic phosphate (P_i_), and CK-facilitated transport operating in the ATP-generating direction (rate constant *k*_*f*_) and PCr-generating direction (rate constant *k*_*r*_). In oxidative muscle under normoxic conditions, cytosolic CK isoforms (CK-MM, -MB, -BB) typically work in the ATP-generating direction, whereas mitochondrial CK works in the PCr-generating direction. Forward CK flux is defined by *k*_*f*_×[PCr], where *k*_*f*_ is the pseudo–first-order unidirectional rate constant in the ATP-generating direction measured using saturation transfer ^31^P magnetic resonance spectroscopy. This should be distinguished from CK total activity (V_Max_), which represents maximal velocity in the presence of saturating amounts of substrate and requires destructive freeze-extraction chemicals. ANT indicates adenine nucleotide transfer; CK, creatine kinase; PCr, phosphocreatine; and VDAC, voltage-dependent anion channel;

Because both reduced CK maximum capacity and CK flux (the rate of total ATP transfer by CK under prevailing conditions in vivo) have been associated with transition to failure in animal models of pressure overload^8–14^ and in human hypertensive hypertrophy^15^ and dilated cardiomyopathy,^16,17^ this may be an important determinant of transition to failure in AS, but this has not been explored.

In addition to CK-facilitated transfer, ATP may also diffuse directly between mitochondria and contractile sites (Figure 1). As such, the distance over which ATP has to diffuse may also affect CK efficacy. In support of this, when CK capacity is impaired in CK knockout models, there is evidence of ultrastructural compensation that effectively reduces this diffusion distance.^18,19^ To date, however, no such measurements exist in human AS. In addition, upstream of ATP transfer, metabolic reserve may be compromised at the level of mitochondrial oxidative capacity, but few human studies assessing phosphotransfer have also measured an index of mitochondrial capacity.

Therefore, this study aimed to use the combination of cardiac magnetic resonance, echocardiography, and biopsy measurements to investigate whether (1) altered CK activity and isoform expression, (2) altered mitochondrial oxidative capacity (measured by citrate synthase activity), or (3) ATP diffusion distance measures on scanning electron microscopy were associated with transition to failure in human SevAS.

## Methods

The data, analytic methods, and study materials will not be made available to other researchers for purposes of reproducing the results or replicating the procedure, because ethical approval to store tissue expires in April 2020 and insufficient tissue remains to replicate results in all patients. All work was approved prospectively by regional (South Central Oxford C, 16/SC/0323) and local ethics and governance committees. All participants were recruited from clinical activity at the Oxford University Hospitals National Health Service Foundation Trust or by poster advertisement. All participants gave formal written consent before all study procedures. Research was performed in accordance with institutional procedures and the principles of the Declaration of Helsinki.

### Inclusion Criteria

Participants were included if they were 18 to 85 years of age and were willing to take part in research. Patients with SevAS were scheduled for aortic valve replacement with either reduced (defined as LVEF <55%) or preserved (defined as LVEF ≥55%) systolic function. Systolic function was determined by cardiac magnetic resonance. Patients with nonhypertrophied, non–pressure-loaded hearts with normal systolic function undergoing cardiac surgery willing to have biopsies taken were also recruited.

### Exclusion Criteria

Participants were excluded if they had known previous myocardial infarction, flow-limiting coronary artery disease, more than mild bystander valve disease, significant renal impairment, pregnancy, or any other contraindication to magnetic resonance imaging scanning. All patients listed for surgery had flow-limiting coronary atheroma excluded by invasive angiography and prior myocardial infarction excluded by late gadolinium enhancement imaging.

On the basis of these criteria, 102 participants were recruited to 5 groups: (1) SevAS, LVEF ≥55% (SevAS with preserved ejection fraction [SevAS-pEF], n=37); (2) SevAS, LVEF<55% (SevAS with reduced ejection fraction [SevAS-rEF], n=15); (3) moderate AS, LVEF >55% ([ModAS], n=13); (4) patients with nonhypertrophied, non–pressure-loaded hearts with normal systolic function undergoing surgery (non–pressure-loaded heart biopsy [NHBx], n=7); and (5) volunteers with non–pressure-loaded hearts with normal systolic function not undergoing cardiac surgery (normal healthy volunteer [NHv], n=30).

### Myocardial Biopsies

Twenty-six patients with SevAS-pEF, 10 with SevAS-rEF, and 7 with NHBx donated intraoperative biopsies sufficiently large to measure creatine and CK total activity. Indications for surgery in biopsied patients with a normal heart were mitral stenosis (n=4), ascending aortic aneurysm (n=1), and benign left atrial mass (n=1). One NHBx sample was not analyzable because of a technical error in the fixing process, leaving 6 for the biopsy analysis in this group.

### Cardiac Magnetic Resonance

All cardiac magnetic resonance was performed on a 3T magnetic resonance imaging scanner (Tim Trio; Siemens) and, unless stated, was analyzed using cvi42 (Circle Cardiovascular Imaging Inc).

#### Left Ventricular Imaging

A short stack of cine images was obtained and analyzed by using a 24-channel spine matrix, 6-channel body matrix, and steady-state free precession sequences as previously described.^20^ In brief, all images were ECG gated and taken during end-expiratory breath-hold. Typical steady-state free precession sequence parameters were slice thickness 8 mm, gap 2 mm, retrospective gating, echo time 1.5 ms, repetition time (TR) 46 ms, flip-angle 50°, field of view 400 mm, and matrix size 256 in frequency encode direction. Prospective gating was used when the participant was in atrial fibrillation. Left ventricular (LV) endocardial and epicardial contours were performed manually in cvi42. LV mass index was normalized to body surface area using the Mosteller formula. Late gadolinium imaging according to clinical protocols was performed to exclude myocardial infarction (further details in Methods in the Data Supplement).

### Cardiac ^31^P Magnetic Resonance Spectroscopy

All magnetic resonance spectroscopy was performed before cardiac magnetic resonance imaging.

#### Phosphocreatine/ATP

Participants were positioned prone over a 3-element dual-tuned ^1^H/^31^P surface coil at magnet isocenter. An 11-minute nongated 3-dimensional acquisition-weighted ultrashort echo time chemical shift imaging sequence was run as previously described.^21^ Parameters included acquisition matrix size 16×16×8 voxels, field of view 240×240×200 mm^3^, nominal voxel size 5.6 mL, 10 averages at the center of k-space, fixed TR per subject (910–1010 ms depending on specific absorption rate constraints), center frequency 250 Hz from phosphocreatine (PCr). The PCr/ATP ratio reported is the blood- and saturation-corrected PCr/average ATP ratio, averaged over the 2 most basal septal voxels. Spectral analysis was performed using OXSA, an open-source MATLAB implementation of the Advanced Method for Accurate, Robust and Efficient Spectral fitting (AMARES) algorithm.^22^

#### Creatine Kinase *k*_*f*_

**Figure 2.**
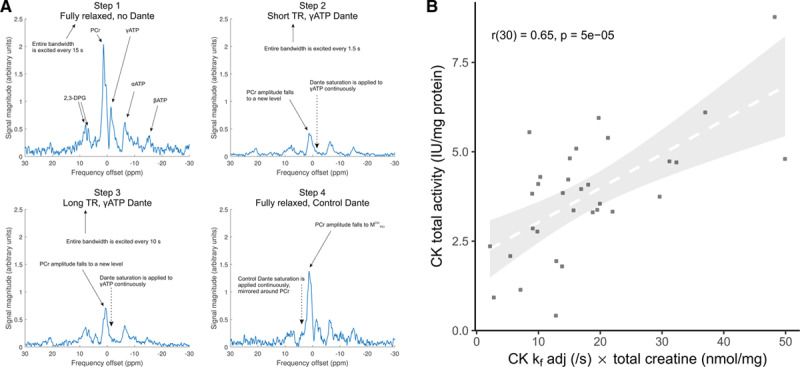
**Validation of noninvasive assessment of CK activity.** **A**, Representative spectra constituting the triple repetition time saturation transfer method for estimating CK *k*_*f*_ in a cardiac voxel of interest. **B**, Absolute CK activity measured from left ventricular biopsy correlated with CK capacity estimated by total creatine×*k*_*f*_ (adjusted for supine scanning). Dante (Delays Alternating with Nutation for Tailored Excitation) is the pulse sequence used for selective saturation of γATP or the control position methods to assess, and in vivo CK capacity, which has been estimated as CK total activity×[total creatine]. CK indicates creatine kinase; 2,3-DPG, 2,3-diphosphoglycerate; PCr, phosphocreatine; and TR, repetition time.

CK *k*_*f*_ was estimated using triple TR saturation transfer adapted for use with a transmit/receive 10-cm loop radiofrequency coil (PulseTeq Ltd) as previously described.^23,24^ In brief, participants were scanned supine; ^1^H localizers confirmed coil position; then a 1-dimensional phase-encoded chemical shift imaging matrix (16 slices, 160 mm) was used to acquire 4 sets of ^31^P spectra. These were a fully relaxed acquisition (TR 15 s, 2 averages, 9 minutes), 2 acquisitions with selective γATP saturation (TR 1.5 then 9.5 s, 18 then 8 averages, 11 then 21 minutes), and 1 with control saturation mirrored around PCr (TR 15 s, 2 averages, 9 minutes) (Figure 2A). Spectral analysis was performed using custom software as previously described.^24^ The pseudo–first-order unidirectional rate constant (*k*_*f*_) of CK in the ATP-generating (forward) direction was calculated according to the following:


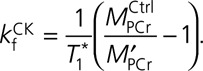


Forward CK flux was calculated by [PCr]×*k*_*f*_, where [PCr] is estimated by multiplying PCr/ATP by literature values for [ATP] (5.7 or 5.2 μmol/g wet weight, the lower value applied to those patients with reduced LVEF).^16^ We multiplied all *k*_*f*_ values by a previously validated factor of 1.333 to adjust for supine scanning.^24^

### Echocardiography

Measurements of the aortic valve were made using standard clinical echocardiography 2-dimensional and Doppler examination of the aortic valve and LV outflow tract (Philips Epiq system). AS was graded on echocardiography according to international guidelines.^25^

### Left Ventricular Biopsies

Surgical myocardial biopsies were obtained from LV endocardium 10 to 20 minutes after starting cardiopulmonary bypass and immediately divided into 2 parts. One part was frozen in liquid nitrogen within 20 seconds of excision and stored at –80°C until analysis; the other was immediately immersed in chemical fixative and further dissected for electron microscopy.

#### Enzyme Activities, CK Isozyme Distribution, and Total Creatine Content

Frozen, powdered samples were analyzed according to established protocols for CK total activity (averaged over 3 runs, normalized to Lowry protein [mg/mL], presented as IU/mg protein), CK isoforms (expression relative to total CK), citrate synthase (CS) activity (averaged over 2 runs, IU/mg protein), and total creatine concentration (nmol/mg protein), as previously described.^26^ (Further details in Methods in the Data Supplement.)

#### Mitochondria-Sarcomere ATP/ADP Diffusion Distance

Samples for serial block-face scanning electron microscopy were fixed and stained with heavy metals for enhanced contrast as previously described,^27^ then dehydrated in a progressive ethanol series, infiltrated with Durcupan epoxy resin, and polymerized at 60°C for 72 hours. Samples were mounted in a Zeiss Merlin Compact Field Emission Gun-Scanning Electron Microscope with Variable Pressure and a Gatan 3View System and underwent serial block-face sectioning (serial block-face scanning electron microscopy) (typical acquisition parameters: magnification 6100×, voltage 3 kV, dwell time 6 ms, pressure 0.3 torr, field of view 40 µm [4000 pixels] square, in-plane [x–y] resolution 10 nm, slice thickness 100 nm). Samples were analyzed in Microscopy Image Browser version 2.0.1 for MATLAB. After applying Perona-Malik anisotropic diffusion filters and grayscale thresholding, manual segmentation was performed over every other slice, to define mitochondria and sarcomeres in a total of 31 slices per data set. For each pixel-labeled sarcomere, an algorithm computed the diffusion distance, defined as the minimum 3-dimensional distance to the nearest pixel-labeled mitochondrion. As such, the diffusion distance was recorded from the mitochondrial edge. Diffusion distance distributions resembled log-normal distributions and were summarized by median distance (see Methods in the Data Supplement).

## Statistical Analysis

Data were reported as means (with SDs) for continuous variables passing Shapiro-Wilk testing for normality and medians (with interquartile range) if not. Box plots depict medians, interquartile range, and whiskers, with whiskers extending to the maximum value of the data within 1.5 times the interquartile range over the third quartile, and the minimum value within 1.5 times the interquartile range below the first quartile.

The relationships between CK total activity and other invasive and noninvasive candidate predictor variables were explored by using linear regression models. In addition, invasive and noninvasive measures between the SevAS-pEF, SevAS-rEF, and NHv and NHBx groups were compared. Continuous variables were compared with parametric tests if the Shapiro-Wilk test and Levene test of between-group homogeneity of variance were passed. The Welch correction was used for unequal sample sizes or if the assumption of homogeneity of variance was violated. If normality assumptions were not met, nonparametric tests were used (Kruskal-Wallis for differences between >2 independent groups, Jonckheere-Terpstra for ordering of medians across >2 groups). *P*≤0.05 was used as a threshold of significance. *P* values were adjusted on post hoc pairwise *t* tests or Wilcoxon rank-sum tests with the Benjamini-Hochberg method to control the false discovery rate. Pairwise comparisons were 2-sided unless otherwise stated. Categorical variables were compared by χ^2^ or, if expected cell counts were <5, by the Fisher exact test. Analyses were performed using R version 3.4.2 (R Foundation for Statistical Computing).

## Results

### Participants

Participant anthropometric and clinical data are shown in Table 1. In the comparison of the NHv, NHBx, ModAS, SevAS-pEF, and SevAS-rEF groups, the proportion of men did not significantly differ, but the median age, body mass index, systolic blood pressure, 6-minute walk distance, history of hypertension, prevalence of left bundle-branch block or ECG LV strain pattern, and the proportion using cardiac medications did.

**Table 1.**
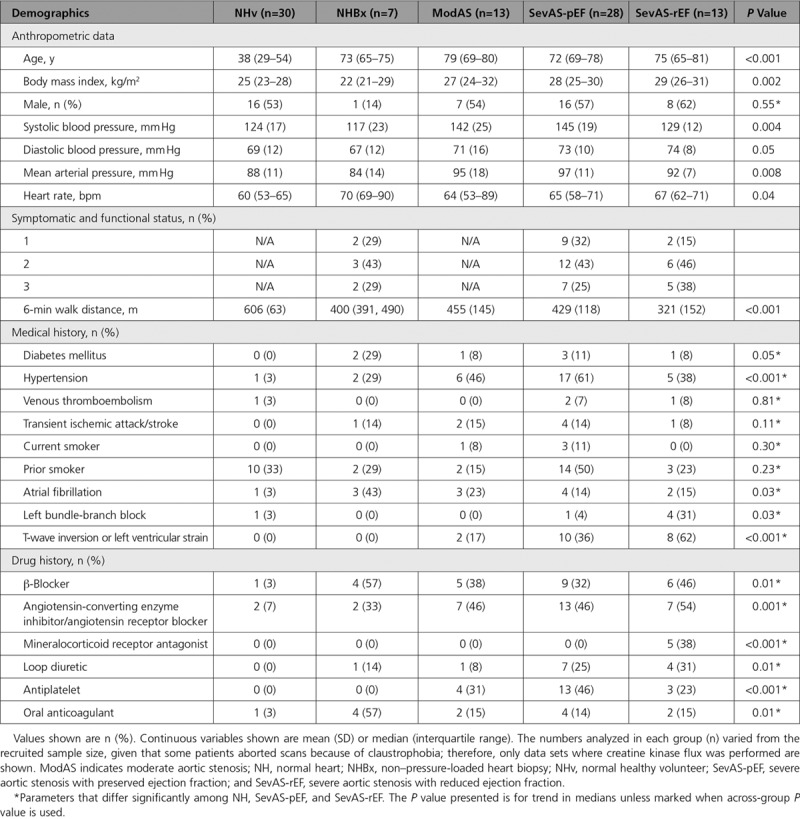
Participants’ Demographics and Medical and Drug History

#### Aortic Valve Assessment

Transthoracic Doppler and 2-dimensional echocardiography measurements of the aortic valve in both SevAS-pEF (V_Max_ 4.4±0.55 m/s, peak gradient 81±20 mm Hg, mean gradient 46±14 mm Hg, aortic valve area calculated by continuity equation 0.83±0.33 cm^2^) and SevAS-rEF groups (V_Max_ 4.1±0.72 m/s, peak gradient 67±26 mm Hg, mean gradient 39±16 mm Hg, aortic valve area calculated by continuity equation 0.62±0.21 cm^2^) confirmed the presence of SevAS. ModAS was confirmed in the ModAS group (V_Max_ 3.3±0.10 m/s, peak gradient 44±8 mm Hg, mean gradient 24±5 mm Hg, aortic valve area calculated by continuity equation 1.2±0.2 cm^2^).

#### LV Characteristics

**Figure 3.**
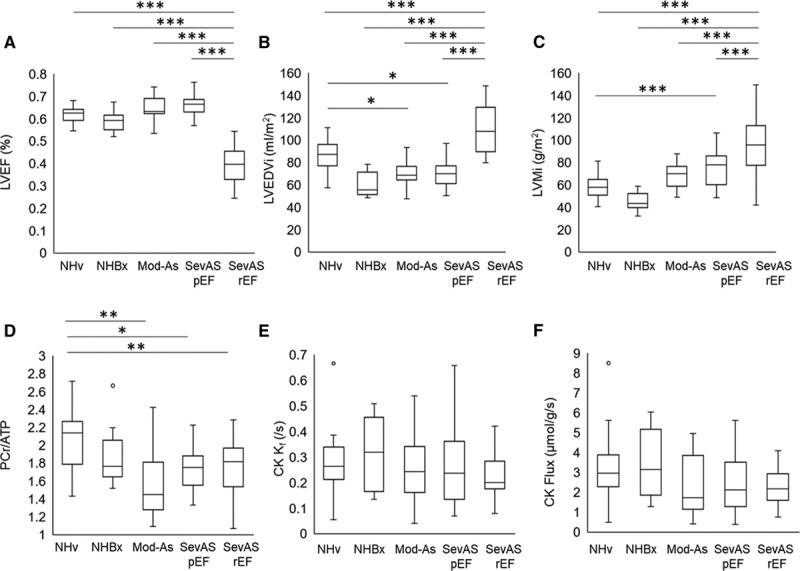
**Cardiac magnetic resonance parameters for the study groups**. LV ejection fraction (**A**). LV end-diastolic volume index (**B**), LV mass index (**C**), magnetic resonance spectroscopy–derived PCr/ATP ratio (**D**), CK *k*_*f*_ (**E**), and forward CK flux (**F**) in nonhypertrophied, non–pressure-loaded healthy volunteer hearts (NHv),in non–pressure-loaded hearts with normal LVEF undergoing LV biopsy (NHBx), and in hearts with moderate AS (Mod-AS), SevAS with preserved (SevASpEF) and reduced (SevASrEF) ejection fraction. For between-group comparison, *P<0.05, **P<0.01, ***P<0.001; otherwise *P*>0.05. AS indicates aortic stenosis; CK, creatine kinase; LVEDVi, indexed left ventricular end-diastolic volume; LVEF, left ventricular ejection fraction; LV, left ventricular; LVMi, left ventricular mass index; and PCr, phosphocreatine.

As expected, because of the recruitment criteria, LVEF was normal in the SevAS-pEF (66±5%), NHv and NHBx groups (61±4%), and ModAS group (65±6%), but significantly lower in the SevAS-rEF group (40±9%, *P*<0.001; Figure 3A). In comparison with the non–pressure-loaded heart groups, mean indexed LV end-diastolic volume (mL/m^2^) was lower in the SevAS-pEF group (by 12%, *P*=0.03) and larger in the SevAS-rEF groups (by 36%, *P*<0.001; Figure 3B). Mean LV mass indexed to body surface area (g/m^2^) was also increased in all SevAS groups in comparison with the non–pressure-loaded groups (Figure 3C). LV mass correlated with New York Heart Association class for those undergoing surgery (LV mass index, *r*=0.29, *P*=0.04).

### CK Flux Validation

To validate the triple TR saturation transfer measurement of CK activity, noninvasive estimates were correlated against ex vivo total CK activity from 32 patients who had undergone both 31P triple TR saturation transfer measurements and intraoperative LV biopsy. When ex vivo CK activity was correlated with triple TR saturation transfer measured in vivo CK *k*_*f*_ (*r*=0.45, *P*=0.01), CK flux (*r*=0.37, *P*=0.04), and CK *k*_*f*_ [total creatine_biopsy_] (*r*=0.65, *P*=5×10^–5^; Figure 2B), there was good correlation between the 2 methods.

### Myocardial Energetics in the Pressure-Loaded LV

To assess whether pressure loading was associated with altered myocardial energetics, all data from participants with AS and normal systolic function (ModAS and SevAS-pEF) were compared with all non–pressure-loaded hearts (NHv and NHBx groups). This showed that pressure loading (peak 71±23 mm Hg) was associated with a 17% lower PCr/ATP (*P*<0.001), no difference in CK *k*_*f*_ (*P*=0.46), and a 32% lower CK flux (*P*=0.04). Reduced CK flux was driven predominantly by reduced PCr pool size (by 17%, *P*<0.001).

#### Myocardial Energetics and the Transition to Failure

To assess whether the altered energetics were associated with transition to failure, ModAS, SevAS-pEF, and SevAS-rEF were compared with the non–pressure-loaded groups.

Myocardial PCr/ATP ratios in all AS groups, including SevAS-rEF, were lower than that recorded in the nonhypertrophied heart (by 16%–21%; Figure 3D). Although PCr/ATP was lower in SevAS-rEF than in the non–pressure-loaded heart, it was not different from SevAS-pEF (*P*>0.99). PCr/ATP was also reduced in ModAS (by 21%, *P*<0.01; Figure 3D). CK *k*_*f*_ was not statistically different between groups (*P*=0.76; Figure 3E).

CK flux was lowest in the failing AS heart (by 27% in comparison with the combined NHv+Bx group), and, when groups were ordered NHBx + NHv > ModAS > SevAS-pEF > SevAS-rEF, this showed that median CK flux decreased as LV mass increased (*P*=0.047; Figure 3F).

Overall this shows that AS is associated with reduced CK flux, with the reduction predominantly driven by a reduced PCr pool size, which is seen at the ModAS stage. It also shows that, although CK flux was lowest in SevAS with systolic failure, energetic depletion appears to precede systolic failure in AS.

#### CK Total Activity

**Figure 4.**
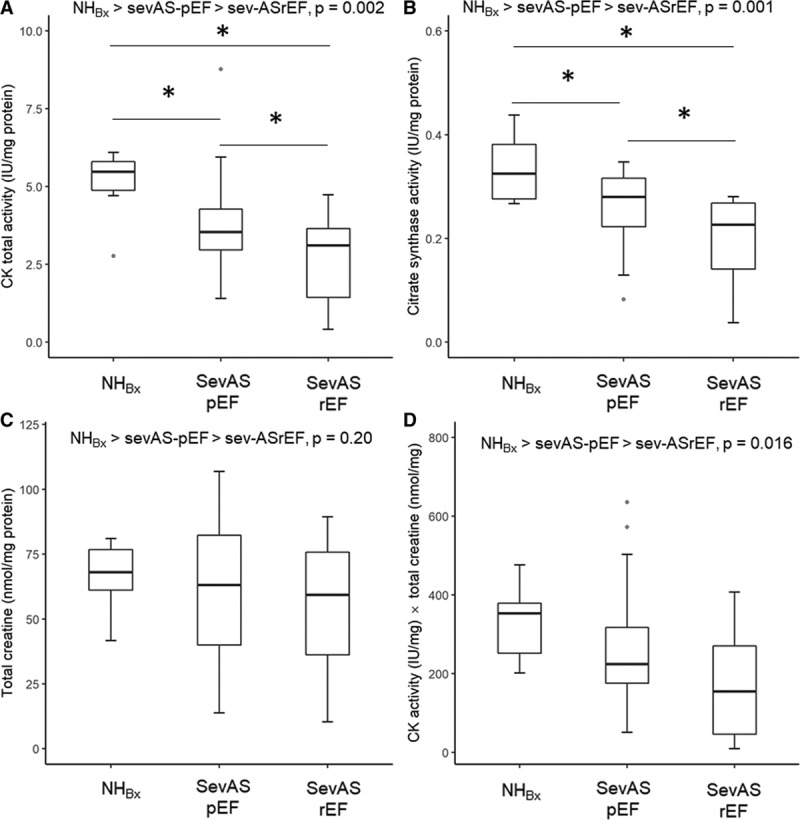
**LV biopsy obtained creatine concentration and CK and CS activities**. CK total activity (**A**), CS activity (**B**), total creatine concentration (**C**), estimated CK capacity (**D**) from nonhypertrophied, non–pressure-loaded hearts (NH), severe aortic stenosis with preserved (SevAS-pEF) and reduced ejection fraction (SevAS-rEF) undergoing valve replacement surgery. *Post hoc pairwise tests significant versus NHBx. †Post hoc pairwise tests significant versus SevAS-pEF. CK indicates creatine kinase; and NHBx, non–pressure-loaded heart biopsy.

In comparison with NHBx hearts, median total CK activity was significantly lower both in SevAS-rEF (by 43%, post hoc one-sided *P*=0.016) and SevAS-pEF (by 35%, post hoc one-sided *P*=0.028), resulting in a decreasing trend in median values (*P*=0.002; Figure 4A, Table 2). Median values were also lower in SevAS-rEF hearts than in SevAS-pEF hearts (post hoc one-sided *P*=0.047).

**Table 2.**
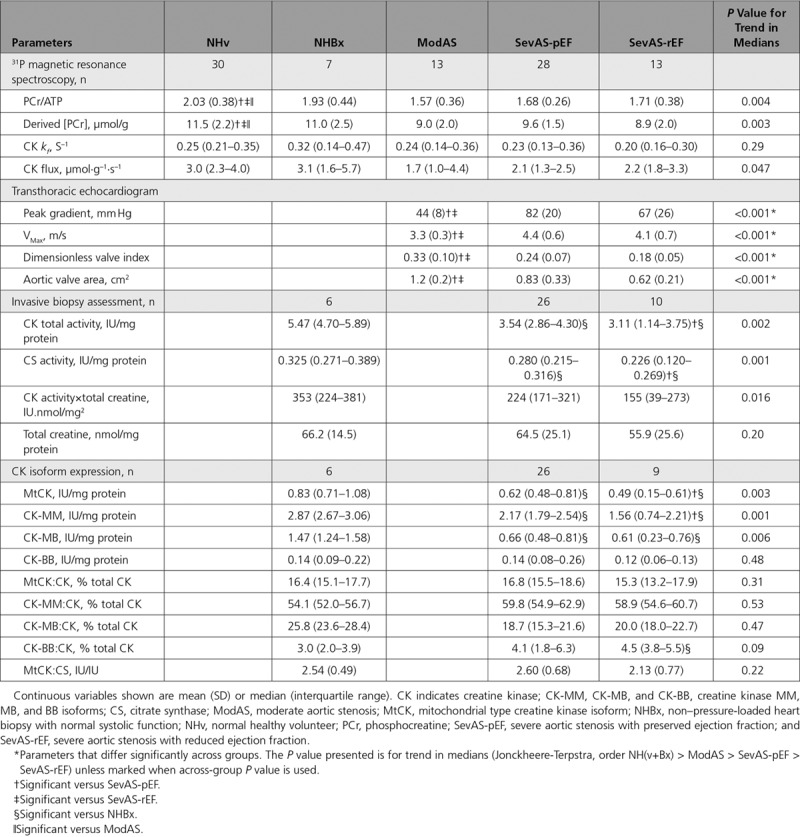
Baseline Energetic Measures and CK Isoform Expression

Forty-two participants (26 SevAS-pEF, 10 SevAS-rEF, and 6 NHBx) contributed data sets eligible for correlation analysis (ie, had measured CK total activity and total creatine), and 32 (19 SevAS-pEF, 8 SevAS-rEF, and 5 NHBx) contributed data sets eligible for linear regression (ie, had measured CK total activity, total creatine, CK *k*_*f*_, and LV mass and volumes). CK total activity correlated strongly with citrate synthase activity (*r*=0.83, *P*=1×10^–11^) and well with total adenine nucleotide pool (*r*=0.50, *P*=2×10^–5^) (Figure I in the Data Supplement, Table I in the Data Supplement). There were good correlations with total creatine concentration (*r*=0.57, *P*=9×10^–5^) and noninvasively estimated CK capacity (*r*=0.65, *P*=5×10^–5^), and moderate correlations with *k*_*f*_ (*r*=0.45, *P*=0.01) and CK flux (*r*=0.38, *P*=0.04) (Figure II in the Data Supplement). There was a moderate positive correlation with LVEF (*r*=0.40, *P*=0.01) and moderate negative correlations with indexed LV end-diastolic volume (*r*=–0.51, *P*=0.001), indexed LV end-systolic volume (*r*=–0.50, *P*=0.001), LV mass index (*r*=–0.44, *P*=0.004), and magnetic resonance imaging–derived global circumferential strain (*r*=–0.52, *P*=0.001) (Figure III in the Data Supplement). Correlations with nonindexed LV volumes and mass were weaker than with indexed counterparts, and not presented. There was no correlation with PCr/ATP ratio. There were only 2 significant correlates of CK *k*_*f*_: CK total activity (*r*=0.45, *P*=0.01) and CK/CS activity ratio (*r*=0.48, *P*=0.005).

#### CS Activity, Total Creatine, and CK Capacity

There was also a significant decreasing trend in median CS activity, an additional marker of oxidative capacity (NHBx > SevAS-pEF > SevAS-rEF, *P*=0.001; Figure 4B), with median CS activity being 30% lower in the SevAS-rEF than NHBx hearts (*P*<0.01), and 14% lower in SevAS-pEF than in NHBx hearts (*P*=0.03). Median values in SevAS-rEF were 19% lower than in SevAS-pEF (*P*=0.02). Comparing total creatine (Figure 4C), there were no significant between-group differences (1-way ANOVA, *P*=0.6) and no decreasing trend in median values (*P*=0.2). A decreasing trend in estimated in vivo CK capacity (CK total activity×total creatine) was also seen (NHBx > SevAS-pEF > SevAS-rEF, *P*=0.016; Figure 4D), but post hoc pairwise tests were not significant.

Overall, this shows that in SevAS with reduced systolic function, both CK activity and CS activity (a marker of cellular oxidative capacity and mitochondrial density) were significantly reduced.

#### CK Isoforms

**Figure 5.**
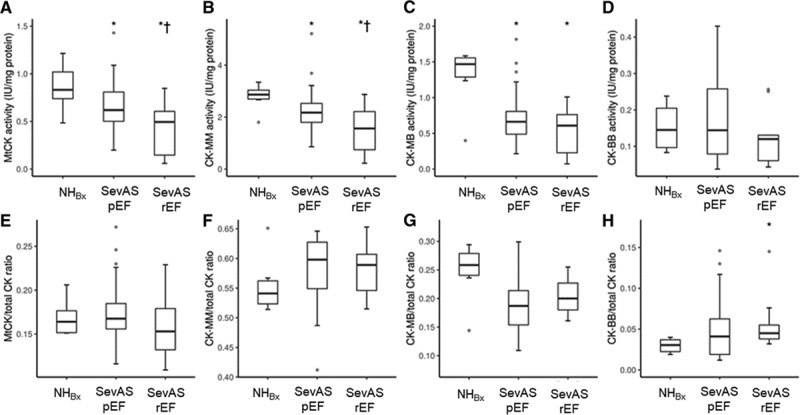
**CK isoform activities in the study groups.** CK isoform activity (IU/mg protein) (**A** through **D**) and CK isoform activity relative to total CK activity (from left to right: MtCK, CK-MM, CK-MB, and CK-BB) in 1 control and 2 AS groups (**E** through **H**). *Post hoc pairwise tests significant versus NH. †Post hoc pairwise tests significant versus SevAS-pEF. AS indicates aortic stenosis; CK, creatine kinase; CK-MM, CK-MB, and CK-BB, creatine kinase MM, MB, and BB isoforms; MtCK, mitochondrial type creatine kinase isoform; NH_Bx_, non–pressure-loaded heart biopsy; SevASpEF, severe aortic stenosis with preserved ejection fraction; and SevAS-rEF, severe aortic stenosis with reduced ejection fraction.

Underlying the observed falls in CK total activity, there were reducing trends in median absolute activities of mitochondrial type CK (MtCK), CK-MM, and CK-MB isoforms when groups were ordered by increasing LV mass index (NHBx > SevAS-pEF > SevAS-rEF, all *P*<0.01; Table 2, Figure 5A through 5D), with post hoc tests significant at each step for MtCK and CK-MM. Absolute activities of CK-MB were also reduced in both SevAS groups, but activities of CK-MB and CK-BB did not differ between the SevAS groups. There were no differences in the relative expression of any isoforms with the exception of CK-BB, whose expression was increased in SevAS-rEF in comparison with NHBx (*P*=0.02) (Figure 5E through 5H). Between-group differences in MtCK/CS activity ratio were not statistically significant.

### ATP/ADP Diffusion Distance

**Figure 6.**
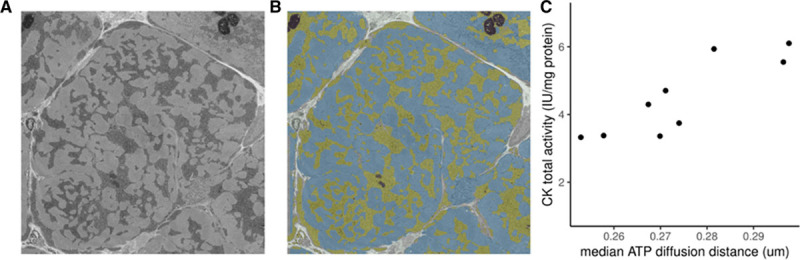
**Relationship between CK activity and ATP diffusion distance.** **A** and **B**, Single unsegmented and segmented scanning electron microscopy slice of a human cardiomyocyte from a patient with severe aortic stenosis with reduced systolic function. Intermediate dark clusters (segmented yellow) represent mitochondria. Magnification 6000×, voltage 3 kV, dwell time 3 ms, pressure 0.188 Torr, image size 4000×4000 pixel (37.6×37.6 µm), slice thickness 100 nm. **C**, Scatterplot of CK total activity against median diffusion distance calculated by 3-dimensional serial block-face scanning electron microscopy. CK indicates creatine kinase.

Nine patients undergoing surgery had sufficient myocardial biopsy size to undergo CK total activity analysis and serial block-face scanning electron microscopy analysis for mitochondrial-sarcomere distances. When median mitochondria-sarcomere diffusion distances were plotted against CK total activity, a strong positive correlation was observed (Figure 6, Pearson *r*=0.86, *P*=0.003) where lower CK activity correlated with shorter ATP diffusion distance. This suggests a compensatory reduction in diffusion distance with lower CK activity.

## Discussion

The transition to systolic failure in AS increases mortality and operative risk. Given the coupling of ATP usage and LV contraction, we investigated the hypothesis that reduced CK capacity/flux is associated with otherwise unexplained systolic failure in human SevAS.

**Figure 7.**
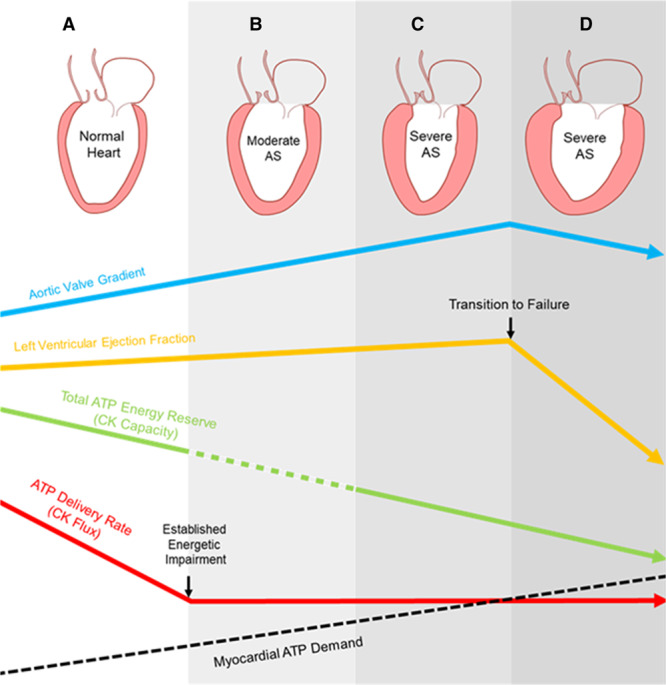
**Central illustration: the effect of pressure loading on myocardial energetics.** In comparison with the normal heart (**A**) energetic impairment occurs in the presence of pressure loading from aortic stenosis (AS) and is established by moderate AS (**B**). Although creatine kinase (CK) capacity reduces (green line) as AS severity increases (blue line) to severe (**C**) and is lowest in systolic failure (**D**), ATP delivery (CK flux, red line) at rest does not alone fall lower than that seen in moderate AS. As ATP demand increases with increasing AS severity and left ventyricular hypertrophy (black dotted line), this exceeds prevailing ATP delivery. In summary, energetic impairment in the CK system is present early in AS, and although it does not appear alone to be sufficient for transition to systolic decline in AS, it may nevertheless increase susceptibility to failure as ATP turnover demands increase.

We show here that, in the presence of SevAS, biopsy-measured total CK activity is reduced and that median values are lowest in those with systolic failure. We also show that there is relative CK isoform redistribution toward CK-MM and CK-BB. In addition, we show for the first time in human pressure overload that cytoarchitectural reorganization occurs when CK activity is lower, resulting in a reduction in mitochondrial to sarcomere diffusion distance. Furthermore, we show that despite a fall in CK total capacity, in vivo magnetic resonance spectroscopy measures of resting CK flux, although again reduced in AS, could not discriminate those with systolic failure, and changes were seen in ModAS. This suggests that significant energetic impairment is already established in ModAS and that reduced CK flux is not by itself necessarily associated with systolic failure, but may predispose to failure in some patients (Central Illustration, Figure 7).

### CK Total Activity

CK total activity represents CK’s maximum ATP transfer capacity and buffering capacity; is an important metabolic reserve, in particular, if cardiac work is increased; and correlates closely with contractile reserve.^5–7^ This suggests that it would be more closely correlated with exercise performance than with resting function in the normal heart. However, if CK total activity falls below the threshold needed to sustain prevailing ATP delivery requirements, systolic dysfunction is expected to occur. Although 2 studies have shown reduced CK total activity in pressure-overloaded human LV,^28,29^ no distinction was previously made between hearts with or without systolic dysfunction. In this study, maximal CK activity from LV biopsy was lowest in SevAS-rEF hearts, in line with noninvasive measures and with previous animal pressure-overload studies.^10,11,14^

### CK Flux

Reduced CK flux has been shown to limit contractile reserve and contribute to the transition to systolic failure in animal studies, but it was unknown whether this is the case in humans. In this study, we have shown that the presence of significant AS was associated with reduced CK flux. Because CK *k*_*f*_ itself was not reduced, this appears to be driven primarily by a reduction in PCr pool and was observed in ModAS. As a result, it seems likely that energetic dysfunction precedes LV systolic dysfunction in pressure loading. In addition, because there was no statistical difference in resting CK flux between SevAS groups with and without systolic failure, this suggests that reduced resting CK flux is not necessary for transition to failure. However, it should be noted that even this normal resting ATP delivery rate may still be inappropriately low, because the ATP demand of the heart increases with increasing AS severity, and the failing pressure-loaded heart becomes more dilated and subject to greater wall stress per gram myocardium. Either this increased ATP turnover must be accounted for by CK-independent means, or, if not, hearts would become more susceptible to transition to failure (Figure 7).

This result is in contrast with the previous report in human hypertensive heart disease,^15^ where PCr/ATP was reduced in hypertensive hypertrophy irrespective of heart failure status, whereas CK *k*_*f*_ was reduced only in the failing group. Given the variability in CK total activity and CK *k*_*f*_ observed here, it is likely that, in AS, CK flux is compromised primarily by a combination of PCr pool depletion and total enzyme activity rather than a fall in CK forward rate constant. Falls in PCr but not CK *k*_*f*_ were previously observed in human failing myocardium after myocardial infarction^30^ and in thoracic aortic constriction models in swine at the nonfailing hypertrophy stage.^11,31^

Overall, these data show that falls in CK flux may appear early in the process of pressure loading and certainly before transition to failure in SevAS. Although these may increase susceptibility to developing systolic failure, a second (and in this study unrecorded) insult is most likely also required.

### CK Isoforms

LV failure has generally been associated with a decrease of myocardial total CK activity and a fetal shift in CK isoform expression secondary to increases in the cytosolic brain type (CK-B) isoforms and decreases of the cytosolic muscle type (CK-M) and mitochondrial (MtCK) isoforms. Congruent with this expectation, we observed stepwise falls in absolute activities of MtCK and CK-MM, a fall in CK-MB absolute activity, and a 50% increase in relative CK-BB expression. The mechanism underlying this isoform shift is unknown. We note that it is not seen in physiological hypertrophy; it is not potentiated by non–B-isoform knockout^32^; it is more marked subendocardially^33^; it is found in multiple heart failure models; and it reverses on unloading.^34^ Therefore, it may relate to chronic wall stress and the fetal isoform shift seen across many genes in pathological hypertrophy.

Most animal models of pressure overload before or during transition to failure found falls in MtCK and CK-M and rises in CK-B (ie, rises in CK-MB and CK-BB) in rats^8,33^ and dogs,^12^ although there are exceptions: in the baboon,^8^ nonfailing thoracic aortic constriction mouse,^13^ and thoracic aortic constriction rat.^35^ In line with the former group of studies, we observed a relative redistribution toward CK-MM and CK-BB. This could be construed as a compensatory adaptation, because CK-MM can functionally couple to various cytosolic ATPases. In addition, CK-BB has greater affinity for PCr and ADP (*K*_m_ of CK-BB for PCr and ADP is 20%–30% and 50%–70%, respectively, that of CK-MM^36^). Thus, for CK-B containing dimers, in comparison with CK-M, the ATP-producing direction is kinetically favored especially at low PCr levels.

### CS Activity

This study also shows a stepwise fall in CS activity when ordered by group (NHBx > SevAS-pEF > SevAS-rEF). Although only 1 marker of oxidative capacity, this suggests that mitochondrial oxidative capacity is reduced in the failing AS heart. In contrast, CS activity was unaltered in animal models of nonfailing pressure overload.^13,34^ The 30% fall in median CS activity in the SevAS-rEF group has no strictly comparable human studies, but in human nonischemic cardiomyopathy both reduced^37–39^ and unchanged activity/expression^40^ have been observed, as has an increase in CS expression after LV assist device implantation.^41^ This suggests that reduced oxidative capacity is also an important consideration in the transition to failure in SevAS. The strong correlation between CK and CS activities observed here would support the argument that the role of CK in myocardium is also primarily associated with oxidative energy production.

### ATP/ADP Diffusion Distance

This study has shown that architectural reorganization occurs in human heart in the setting of reduced CK flux. Although it has been argued that these changes are physiologically unimportant given that the median diffusion distance values measured (0.26–0.29 µm) are 1 to 2 orders of magnitude smaller than noninvasive estimates of PCr diffusion path length in skeletal muscle,^42^ it has been shown in CK knockout models^18^ that diffusion distances also shorten. In the CK knockout model, this shortening is hypothesized to facilitate CK-independent metabolite channeling, which can be extended to human AS given the findings in this study. In addition, the retarding effect of membrane barriers could magnify the effect of small changes in diffusion distance making these small changes in distances meaningful. Although this study cannot establish the physiological importance of this architectural reorganization, it highlights that it occurs in both rodent CK knockouts and human cardiac pressure loading. Thus, changes in CK activity do not appear to occur in isolation, and architectural remodeling may occur to facilitate ATP metabolism.

### Limitations

These results do not establish the timing of any falls in CK and CS activity in relation to AS severity or indeed LVEF trajectory, and so it is not possible to infer causal relationships. To answer this would require within-person repeated sampling, which is infeasible given the requirement for LV biopsy. Nor can we establish the effect of baseline CK activity on LVEF trajectory, which would require longitudinal design. We did not assess the CK system under conditions of acutely increased stress, when the CK system is thought to be most important, because contractile reserve and stress testing are contraindicated in symptomatic SevAS. We also did not measure CK-independent phosphotransfer through adenylate kinase. Adenylate kinase contributes ≈10% of phosphotransfer in the normal heart and can increase its contribution in heart failure (up to 21% in dogs), albeit not sufficiently to fully compensate for large changes in the CK system.^43^ Our estimate of CK flux was based on literature values of [ATP] because absolute quantification was not completed.

Mitral stenosis does not represent the truly normal heart, but was considered the best compromise source of ethically accessible, nonischemic, non–pressure-loaded, similarly aged comparator human LV.

CS activity was measured as a marker of oxidative capacity. Although CS activity is only a surrogate marker of oxidative capacity,^44,45^ we feel that it was the only reliable way of getting a readout of oxidative capacity given the small (20–30 mg) LV biopsy sample size.

We did not measure classical markers of pathological hypertrophy (atrial natriuretic peptide, α-actin, myosin heavy chain) or myocardial fibrosis, because scan time and biopsy sizes were already at the upper limit of acceptable tolerance. Biopsy size also precluded a polarographic assessment of oxidative capacity. It is also possible that there were patients transitioning to failure in the (mostly symptomatic) SevAS-pEF group, which could downwardly bias CK activity values. Conversely, the sickest patients with SevAS-rEF could neither be recruited nor biopsied, because they either felt too unwell to consent or were not listed for surgical aortic valve replacement. This could exert upward bias on values recorded.

## Conclusion

Why some but not all patients with SevAS develop otherwise unexplained reduced systolic function is unclear. This study shows that total CK capacity is reduced in the presence of SevAS and is lowest in those with systolic failure. Despite this, in vivo measures of resting CK flux, although lower in AS, could not discriminate those with systolic failure. In addition, we show that energetic impairment is established in the earlier clinical stage of ModAS. This suggests that significant energetic impairment develops early in AS and is not a necessary correlate of systolic failure, but it could make patients more susceptible to systolic decline because ATP demands increase with progressive increases in AS severity. Overall, these results provide a better understanding of the energetic mechanisms that underlie the transition to failure in AS and should stimulate the development of new therapeutic strategies in this area, for example, improving cardiac metabolism to delay transition to failure. Whether noninvasive metabolic imaging techniques could be used to identify those at risk of transition to failure is worthy of further investigation.

## Acknowledgments

The authors thank Professor P.A. Bottomley for his academic input on this project. Electron microscopy work was performed at the Dunn School EM Facility, and the authors thank R. Dhaliwal, A. Fyfe, and A. Pielach for preparing samples for serial block-face scanning electron microscopy.

## Sources of Funding

This study was principally funded by a British Heart Foundation Clinical Training Research Fellowship FS/15/80/31803 (to Dr Peterzan) with support from a British Heart Foundation Program Grant (RG/18/12/34040). Drs Neubauer and Rider acknowledge support from British Heart Foundation Center of Research Excellence. Dr Neubauer acknowledges support from the National Institute of Health Research Oxford Biomedical Research Center. Dr Rodgers receives funding from the Wellcome Trust and the Royal Society (grant no. 098436/Z/12/B) and supported by the National Institute of Health Research Cambridge Biomedical Research Center. Dr Rider is funded by the British Heart Foundation FS/16/70/32157. Dr Miller was supported by a Novo Nordisk Postdoctoral Fellowship run in conjunction with the University of Oxford. The Biotechnology and Biological Sciences Research Council provided Advanced Life Sciences Research Technology Initiative 13 funding for serial block-face scanning electron microscopy through grant BB/C014122/1 (to Prof Chris Hawes, Oxford Brookes University).

## Disclosures

The views expressed are those of the author(s) and not necessarily those of the UK National Health Service, the UK National Institute of Health Research, or the UK Department of Health and Social Care.

## Supplemental Materials

Methods

Data Supplement Figures I–III

Data Supplement Table I

References 46–48

## Supplementary Material


