# Supplementary material for: Cardiac Energetics in Patients With Aortic Stenosis and Preserved Versus Reduced Ejection Fraction
Source: Circulation. 2020 May 22;141(24):1971–85. doi: 10.1161/CIRCULATIONAHA.119.043450 (PMC7294745; doi:10.1161/CIRCULATIONAHA.119.043450)
Supplement: Supplementary file 1 [file cir-141-1971-s001.pdf]

Supplemental Material

Supplemental Methods

Cardiac ^{31}P-magnetic resonance spectroscopy

PCr/ATP

Participants were positioned prone over a three-element dual-tuned $^{1}H/^{31}P$ surface coil at magnet isocentre. An 11 min non-gated 3D acquisition-weighted ultra-short echo time CSI sequence was run as previously described[21]. Parameters included: acquisition matrix size 16 × 16 × 8 voxels, field of view 240 × 240 × 200 mm^3, nominal voxel size 5.6 ml, 10 averages at the centre of k-space, fixed TR per-subject (910-1010 ms depending on specific absorption rate constraints), centre frequency 250 Hz from PCr. The PCr/ATP ratio reported is the blood- and saturation-corrected PCr/average ATP ratio, averaged over the two most basal septal voxels. Spectral analysis was performed using "OXSA", an open-source MATLAB implementation of the AMARES algorithm[22].

CK k_f

CK k_f was estimated using an implementation of triple repetition time saturation transfer (TRiST) modified to suit the 10 cm transmit-receive circular RF coil (PulseTeq Ltd, Chobham, UK) and multinuclear 3T scanner (Trio; Siemens, Erlangen, Germany) available at our centre[23,24]. With participants positioned supine for approx. 1 hour, we acquired transaxial and sagittal ^{1}H localisers to confirm coil position over the apical LV septum and at magnet isocentre (repositioning until satisfied), free induction decay (FID) inversion recovery scans of a phenylphosphonic acid (PPA) fiducial to calibrate transmit field (B_1^+), imaging of cod-liver oil position markers (to calibrate coil position), and a transaxial slice level with the coil centre (for voxel placement). A 1D phase-encoded CSI matrix (16 slices, 160 mm) was oriented coronally with sixteen 1 cm-deep voxels aligned parallel to the chest wall, aiming to place two voxels anterior to the chest surface and the voxel of interest (VOI) over the most apical unambiguously cardiac voxel.

A 5 min frequency-finding sequence (1D-CSI, 16 phase encode steps for depth selectivity, TR 330 ms, TE 2.3 ms, flip-angle 35º, 125 averages, centre frequency 250 Hz negative to PCr) was run with a 25 mm saturation band placed over chest wall skeletal muscle. This was analysed in MATLAB on a

computer adjacent to the scanner console to generate expected centre frequencies and frequency offsets of each of the 16 voxels. Those for the VOI were programmed for the four TRiST acquisitions. These comprised a fully-relaxed acquisition (TR 15 s, 2 averages, 9 min), two with selective γATP saturation (TR 1.5 then 9.5 s, 18 then 8 averages, 11 then 21 min), and one with control saturation mirrored around PCr (TR 15 s, 2 averages, 9 min), with pulse sequences and spectral analysis as described previously[24] generating a 1D coronal stack of k_f values. The value in the VOI was exported for group statistical analyses. To allow comparison with published literature k_f was adjusted for the effects of our supine coil position (vs prone in previous studies) by multiplying by 1.333 (as described previously)[24].

Biochemical analysis of LV biopsies

10-20 min after going on cardiopulmonary bypass, surgical myocardial biopsies were obtained from LV endocardium and immediately divided into two parts. The larger part was frozen in liquid nitrogen within twenty seconds of excision and the smaller, a thin sample (roughly 0.5 × 1 mm), was bathed in primary fixative and further divided into parts three times smaller for electron microscopy. The larger part was stored at -80 ºC until analysis. All biochemical analyses were performed on ice unless otherwise specified.

Samples were crushed into powder using a metal pulveriser precooled with dry ice. A heaped spatula-full of frozen, crushed powdered LV (5–10 mg) was reconstituted in a droplet of ice-cold homogenizing buffer (K_2HPO_4 0.08 M, KH_2PO_4 0.02 M, β-mercaptoethanol 1 mM, EGTA 1 mM, pH 7.4), excess liquid chased off and the weight of tissue recorded. The sample was placed in a glass homogenising vessel with additional ice-cold homogenisation buffer, with the buffer volume adjusted to achieve a tissue concentration in buffer of 5 mg/ml. The sample was then homogenised using a stirrer at 1300 rpm for 30 sec. A 200 µl aliquot of the homogenate was mixed with 200 µl of 4% NaOH to create a solution for the Lowry assay. This solution was heated in a water bath at 60ºC for two hours then stored at 4ºC until analysis. The remainder was settled for 30 min at 4ºC with 0.1% Triton-X 100 to permeablize membranes. The resulting supernatant was used for subsequent measurements.

CK activity

Supernatant was diluted 1:5 in ice-cold homogenization buffer and kept on ice. CK-NAC reagent (catalogue code TR14010, Thermo Fisher Scientific) was warmed to 30ºC in a water bath. At the point of measurement, 20 µl of diluted supernatant was placed in a cuvette and 1 ml CK-NAC was added. The rate of increase of absorbance of NADPH at 340 nm and 37ºC, which is proportional to CK activity, was then monitored over 10 min using a spectrophotometer. CK velocity (IU/ml) was calculated from the rate of change in absorbance of NADPH, corrected for reaction volume and an assay-specific correction factor, averaged over three runs and normalised to Lowry protein (mg/ml). Results were presented as CK velocity (IU/mg protein).

CK isoforms

5 µl of supernatant was diluted 1:20 with ice-cold homogenization buffer and incubated with SPIFE CK vis activator (SAS-1+ kit, cat. no. 3332, 3333, Helena Biosciences) at room temperature for 10 min. The mixed CK isoenzyme reagent underwent agarose gel electrophoresis using SAS-1+ kits to separate CK isoenzymes based on electrophoretic mobility. The separated isoenzyme bands were visualised by adding isoenzyme reagent and isoenzyme chromogen to the gel after 30 min. The gel was then incubated (60ºC, 20 min), destained (0.3% acetic acid, 10 min), washed, dried (60ºC, 30 min) and scanned (ChemiDoc MP Imager, Bio-Rad). The relative concentration of bands was quantified by densitometry.

Citrate synthase activity

50 µl of supernatant was incubated with 850 µl of reagent (acetyl-CoA 0.35 mM, 5,5'-dithio-bis(2-nitrobenzoic acid) (DTNB) 0.12 mM; all solutions made up in Tris base adjusted to pH 8.1) for 3 min at room temperature. 100 µl of 10 mM oxaloacetate was added and absorbance at 412 nm monitored over 1 min. Mean activity over 40 s and two runs was reported in IU/mg protein.

Total creatine concentration

'Standards' containing known standard concentrations of creatinine, creatine, PCr, AMP, ADP and ATP were run through the HPLC column (SupelcosilTM LC-18-T, 5 umol, cat. no. 58971, Sigma Supelco) at three dilution strengths and their concentrations plotted against peak area under the chromatogram to generate calibration curves for each molecule.

Frozen crushed LV was homogenised in perchloric acid, neutralised with potassium hydroxide, adjusted to pH 7, centrifuged at 4ºC and filtered. All preparation was done on ice. 10 ul of homogenate was loaded onto the HPLC column under standard conditions (28ºC, flow rate 0.7 ml/min, detection wavelength 206 nm, mobile phase 3.5% acetonitrile, 215 mM potassium dihydrogen orthophosphate, 2.3 mM tetrabutylammonium bisulphate). Concentrations (nmol/ml) were normalised to Lowry protein (mg/ml) and presented as nmol/mg protein.

Lowry protein concentration

The solution for the Lowry assay was diluted in triplicate in water (1:10 for CK and CS assays, 1:50 for creatine assays). Duplicate control 'standards' were made using known concentrations of BSA 0-80 µg/ml. Samples and standards were mixed with 1 ml Lowry reagent, vortexed and incubated at room temperature for 20 min. 500 µl Folin and Ciocalteu's phenol reagent was added and samples were vortexed and incubated at room temperature for 30 min. Samples were then filtered into cuvettes (3 ml syringe, 0.45 µm filter) and protein concentration quantified by measuring absorbance at 750 nm.

Serial block-face scanning electron microscopy (SBF-SEM)

Sample fixation and imaging

The purpose of the SBF-SEM was to generate 3D sarcomere-mitochondrion distance maps for each dataset, which would represent diffusion distance distributions for high-energy phosphates. This influenced the choices and compromises made below, in particular: resolution was not optimised for assessment of mitochondrial number or health; alternate slices were analysed rather than single slices to maximise 3D coverage.

Newly separated biopsies were transferred to freshly prepared primary fixative (2.5% glutaraldehyde + 4% formaldehyde in 0.1M sodium cacodylate buffer, pH 7.4) as soon as possible then stored at 4ºC for up to four weeks before batch processing. Samples were then washed and underwent secondary fixation A with osmium ferricyanide (1.5% potassium ferricyanide + 2% osmium tetroxide in 0.1M sodium cacodylate buffer). Samples then underwent further washing and staining cycles with thiocarbohydrazide, 2% osmium tetroxide (secondary fixation B), 1% uranyl acetate (overnight tertiary fixation), and lead aspartate, followed by dehydration cycles with increasing concentrations of

anhydrous ethanol (30–100%) and 100% acetone (day 2). After dehydration, samples were infiltrated with Durcupan epoxy resin (with acetone as the transition solvent) overnight and incubated in a microwave processing unit (Leica AMW), then embedded in flat bottom Beem capsules in fresh Durcupan resin and polymerised at 60 °C for 72 hrs.

Acquisition parameters were determined by experimentation, aiming to achieve sufficient resolution to identify mitochondrial outlines but balancing this against achieving a wide field of view and acceptably short time per slice. Pressure was tuned to reduce charging of the sample surface. Typical acquisition parameters (magnification 6100×, voltage 3 kV, dwell time 6 ms, pressure 0.3 Torr, field of view 40 µm square or 4000 × 4000 pixels, slice thickness 100 nm, pixel pitch 10 × 10 nm) resulted in approximately 220 slices serially sectioned and imaged over 24 hours for each dataset. [46-48]

Sample analysis

Manual segmentation was performed over 30 alternate slices to improve the existing model, aiming to define areas as mitochondria, sarcomere or neither. Due to the time-intensive nature of the task and the redundancy within the data (the typical z-dimension of a mitochondrion spanned ~5-6 consecutive slices), alternate- and consecutive-slice segmentation strategies were compared for one dataset. As the results were similar, an alternate-slice approach was taken. Each dataset required 7-21 working days to manually segment to a satisfactory standard. [46-47]

After manual segmentation, the shortest distance for any pixel labelled sarcomere to the nearest pixel labelled mitochondrion in 3D, here called diffusion distance, was computed across the 3D dataset using a MATLAB script supplied by Dr Ilya Belevich. Raw histograms of the diffusion distances displayed modal spikes at multiples of the inter-slice z distance, reflecting an artefact of the voxel dimension ratio (1:1:10). To mitigate this, a linear interpolation script (MATLAB’s “interpmask”) was run on both the stack of 8-bit tiffs and the manually segmented model, such that four synthetic slices were interpolated between each pair of consecutive slices (5-fold interpolation for tiffs). Similarly, 10-fold interpolation was run on the model in the z-direction and 0.5-fold interpolation in the xy plane, resulting in isotropic 20 nm voxels and so reducing the matrix size from 4000 × 4000 × 4000 to 2000 × 2000 × 301. Diffusion distance distributions were thus smoothened. While resembling log-normal distributions, no single parameter could perfectly summarise the distributions, and median distance was reported.

Supplemental Tables

Supplemental Table I. Invasive and non-invasive correlates of CK total activity.

Parameter	N	Pearson r	p-value (r)	Spearman rho	p-value (rho)
CS total activity (IU/mg protein)	42	.83	1×10^{-11}	.83	2×10^{-16}
CK/CS activity ratio (IU/IU)	42	.61	2×10^{-5}	.52	5×10^{-4}
TAN pool (nmol/mg protein)	25	.50	.012	.50	.012
Total creatine (nmol/mg protein)	42	.57	9×10^{-5}	.58	8×10^{-5}
CK k_f adj (/s)	32	.45	.009	.33	.070
k_f adj × estimated [PCr] (μmol/g/s)	30	.37	.044	.38	.041
k_f adj × total creatine (nmol/mg/s)	32	.65	5×10^{-5}	.58	7×10^{-4}
LVEDVi (ml/m^2)	41	–.51	6×10^{-4}	–.55	3×10^{-4}
LVESVi (ml/m^2)	41	–.50	9×10^{-4}	–.44	.005
LVEF (%)	41	.40	.011	.30	.053
LVMi (g)	41	–.44	.004	–.47	.002
MRI global circumferential strain (%)	39	--.52	7×10^{-4}	--.45	.004

Supplemental Figures

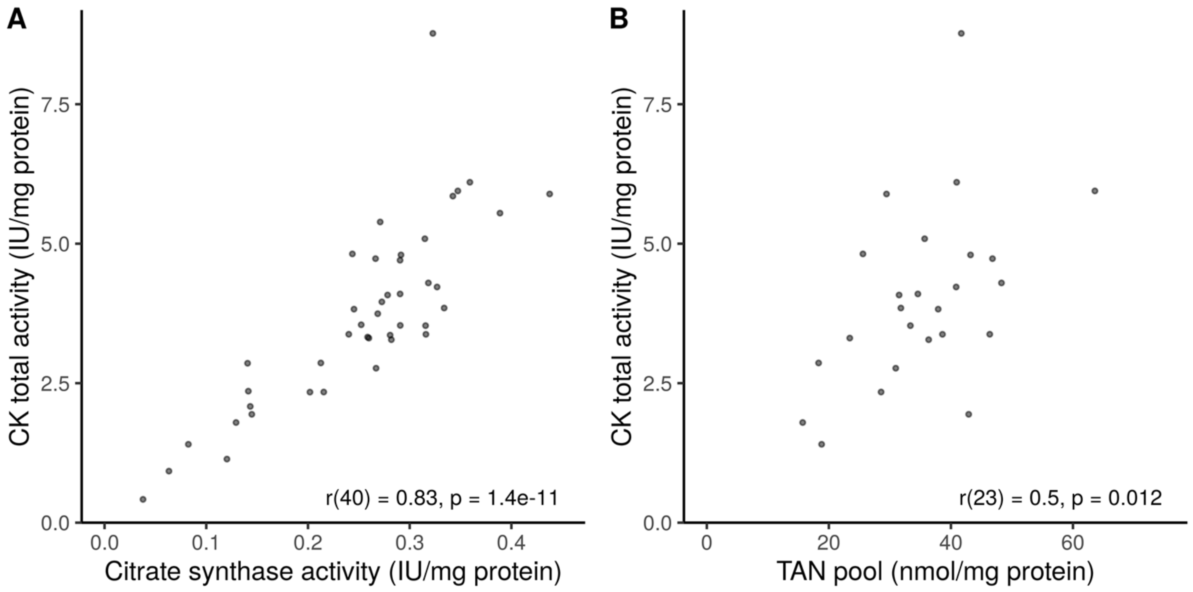

Supplemental Figure I. Scatter plots for (A) citrate synthase (CS) activity, (B) total adenine nucleotide (TAN) pool against CK total activity.

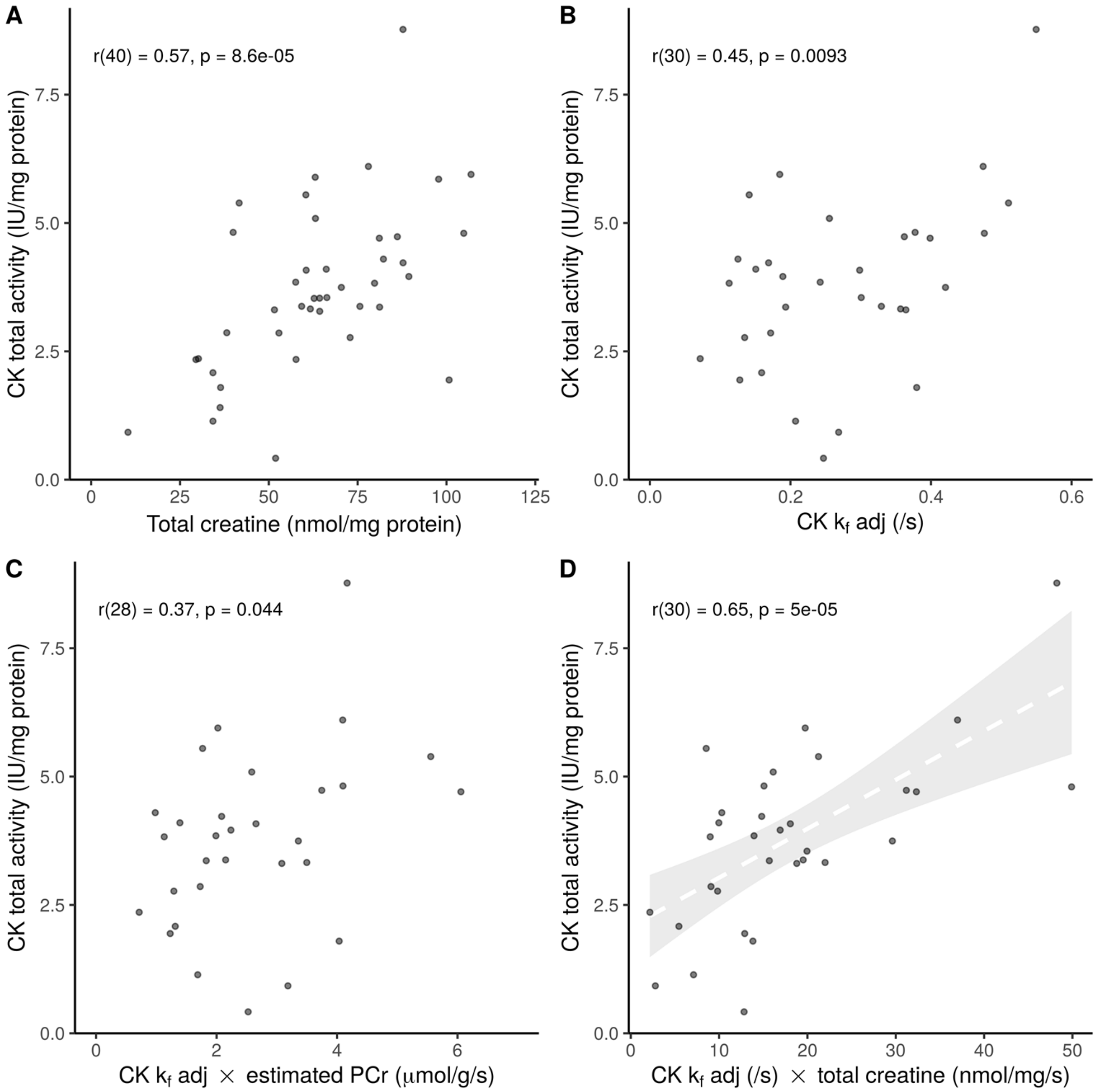

Supplemental Figure II. Scatter plots for (A) total creatine, (B) CK k_f (adjusted for supine position), (C) CK flux estimated by k_f × estimated [PCr], and (D) CK flux estimated by total creatine × k_f against CK total activity.

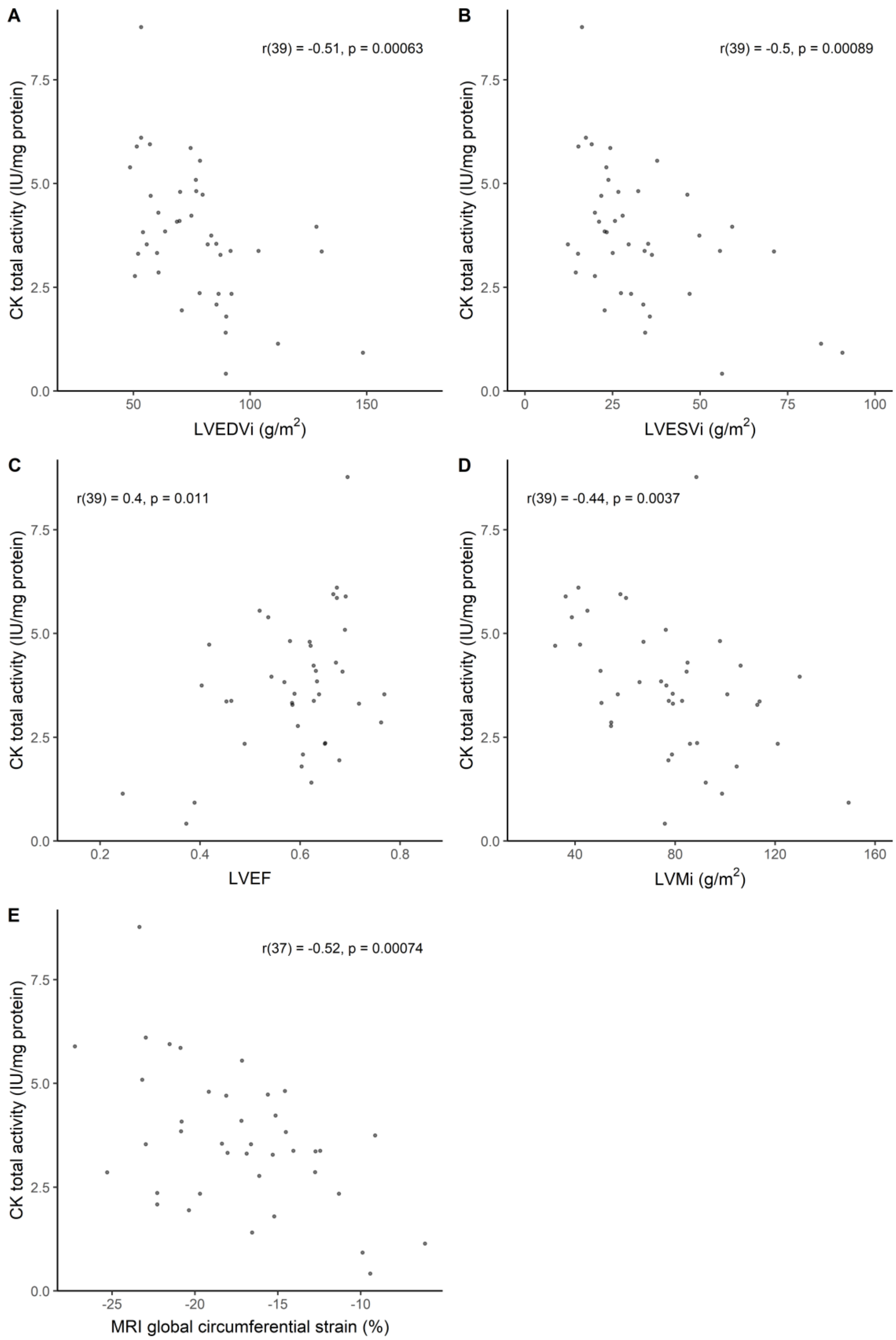

Supplemental Figure III. Scatter plots for (A) left ventricular end-diastolic volume index, (B) left ventricular end-systolic volume index, (C) left ventricular ejection fraction, (D) left ventricular mass index, and (E) MRI global circumferential strain against CK total activity.
